# Overcoming Barriers to Skills Training in Borderline Personality Disorder: A Qualitative Interview Study

**DOI:** 10.1371/journal.pone.0140635

**Published:** 2015-10-14

**Authors:** Kirsten Barnicot, Laura Couldrey, Sima Sandhu, Stefan Priebe

**Affiliations:** Unit for Social and Community Psychiatry, Department of Medicine, Queen Mary University of London, London, United Kingdom; Central Institute of Mental Health, GERMANY

## Abstract

Despite evidence suggesting that skills training is an important mechanism of change in dialectical behaviour therapy, little research exploring facilitators and barriers to this process has been conducted. The study aimed to explore clients’ experiences of barriers to dialectical behaviour therapy skills training and how they felt they overcame these barriers, and to compare experiences between treatment completers and dropouts. In-depth qualitative interviews were conducted with 40 clients with borderline personality disorder who had attended a dialectical behaviour therapy programme. A thematic analysis of participants’ reported experiences found that key barriers to learning the skills were anxiety during the skills groups and difficulty understanding the material. Key barriers to using the skills were overwhelming emotions which left participants feeling unable or unwilling to use them. Key ways in which participants reported overcoming barriers to skills training were by sustaining their commitment to attending therapy and practising the skills, personalising the way they used them, and practising them so often that they became an integral part of their behavioural repertoire. Participants also highlighted a number of key ways in which they were supported with their skills training by other skills group members, the group therapists, their individual therapist, friends and family. Treatment dropouts were more likely than completers to describe anxiety during the skills groups as a barrier to learning, and were less likely to report overcoming barriers to skills training via the key processes outlined above. The findings of this qualitative study require replication, but could be used to generate hypotheses for testing in further research on barriers to skills training, how these relate to dropout, and how they can be overcome. The paper outlines several such suggestions for further research.

## Introduction

Dialectical behaviour therapy (DBT) has been found effective in numerous randomised controlled trials for reducing suicide and self-injury, and for improving global outcomes in borderline personality disorder (BPD) [1, 2, 3].

DBT is based on the biosocial theory of BPD. A major premise is that BPD develops when emotionally sensitive individuals encounter invalidating environments that ignore, suppress or punish their emotions, which further compounds their emotional sensitivity and prevents development of the behavioural and cognitive skills required to self-regulate emotions. DBT therefore has five essential functions: 1) to teach skills for more effective emotional and behavioural regulation, 2) to enhance client motivation to use these skills, 3) to ensure clients can use the skills in a wide variety of situations, 4) to help shape an environment that reinforces skill use and 5) to enhance the therapist’s own skills and motivation to keep working effectively with the client. These functions are fulfilled via five modes: 1) group skills training sessions, 2) individual therapy, 3) telephone skills coaching, 4) case management and 5) therapist consultation meetings [4,5].

The frequency with which clients use the DBT skills is positively associated with self-harm reduction [6,7] improvement in other features of BPD [8] and completing treatment [6]. In qualitative interviews, many clients have reported finding the DBT skills helpful for regulating their emotions and behaviour [9, 10, 11, 12]. However, little research on barriers to treatment progress in DBT has been conducted [13,14]. Such research is important in order to optimise treatment delivery and clinical outcomes [15, 16]. Given the evidence base for skills training as a treatment mechanism, it may be particularly important to explore what factors facilitate or act as barriers to DBT skills training. Qualitative interviews with clients who have received DBT could provide a rich source of experiential data, whilst comparing experiences between treatment completers and treatment dropouts could enable generation of hypotheses about processes contributing to dropout.

The present study therefore used thematic analysis of qualitative interviews with clients receiving DBT to address the following questions:

1. What factors do clients experience as barriers to DBT skills training?
2. How do clients experience overcoming barriers to skills training?
3. How do experiences of barriers to skills training, and overcoming such barriers, differ between treatment completers and dropouts?

## Method

### Inclusion Criteria

The inclusion criteria were:

1. Diagnosis of BPD
2. Having initiated in the past twelve months a twelve-month course of DBT, consisting of once-weekly individual therapy, once-weekly group skills training, telephone skills coaching and team consultation.

All participants were concurrently taking part in quantitative evaluations of DBT [6, 17], which required a recent history of self-harm (past 12 months).

### Measures

Participants’ Axis I and Axis II diagnoses were established at the beginning of treatment using the Mini International Neuropsychiatric Interview [18] and the Structured Clinical Interview for DSM-IV Axis II (SCID-II) [19]. Frequency of self-harm in the twelve months before beginning DBT was established using a structured interview based on the definitions of self-harm used in the Suicide and Self-Injury Interview [20], whilst BPD severity was assessed using the Zanarini Scale for BPD [21]. These measures were administered in the course of quantitative evaluations of DBT, in which all participants were concurrently participating, separately to the current study [6, 17].

### Recruitment and Sampling

Forty interviewees were purposively sampled from a group of 89 individuals participating in a process-outcome study of DBT [6], some of whom were also concurrently participating in a randomised controlled trial [17], between 2009 and 2012. The purposive sampling [22] selected both treatment completers and treatment dropouts to be interviewed, where treatment dropouts were those missing four or more consecutive sessions of group and/or individual therapy [5]. This aimed to facilitate maximum variation sampling [22], whereby a range of experiences was captured, and to enable comparison of treatment completers and dropouts. Completers were consecutively selected to be interviewed once they had completed treatment, until data saturation had been achieved i.e. a subjective judgement by the study authors that additional interviews were not contributing new ideas to the analysis [23]. Selection of dropouts for interview was also consecutive until the point of data saturation, but some dropouts lost contact with the research team before they could be interviewed. More completers were sampled (N = 27) than dropouts (N = 13). This was because completers by definition had lengthier and more complex experiences of skills training to draw on, so saturation took longer to achieve in this subgroup.

### Study Setting

#### Sample Characteristics

The sample was predominantly female (85%) and unemployed (67.5%), and spanned a range of ethnic groups (55% white, 45% black or other ethnic minority). The average age was 33 years (*SD* = 10.2). On average participants met criteria for 3 personality disorders (*SD* = 1.3) including BPD, and co-morbidities included substance dependence (33%), alcohol dependence (36%) and post-traumatic stress disorder (50%). In the 12 months before beginning DBT, the average rate of self-harm was 7.6 days (*SD* = 10.1) per month. The treatment completer subgroup (N = 27) had all completed 12 months of DBT, whilst the treatment dropout subgroup (N = 13) had completed an average of 6.1 months (*SD* = 3.2, range: 1–11 months).

#### Treatment Setting

Participants were recruited from a community DBT service in an inner city area in the United Kingdom. The service provided a twelve-month course of DBT, including weekly individual therapy, a weekly group skills teaching session, telephone skills coaching and team consultation. Since this paper focuses on barriers to treatment, it is important to establish treatment adherence. A DBT therapist was trained in adherence rating by the Behaviour Research and Therapy Clinics (BRTC), University of Washington, and rating was conducted according to BTRC protocol. All individual and group sessions were audio recorded. Ten percent of individual sessions and five group sessions were randomly selected for adherence rating. A further 10% of the selected individual sessions and one group session were also rated by a BRTC adherence rater to establish inter-rater reliability. The individual sessions received a mean score of 4.1 (range: 3.6–4.5), demonstrating good adherence. The skills teaching groups received a mean score of 4.1 (range: 4.0–4.4). The two raters rated all sessions within a 0.3-point margin of difference.

#### Interview Setting

Participants were interviewed as soon as possible after ending DBT. The interviews were conducted, following informed consent, in a private research office or the client’s home, lasted 20 to 90 minutes and were audio recorded for later transcription.

### Reflexivity

The background, research roles and potential influences on each of the authors is detailed in Table 1. The authors aimed to approach data collection and analysis without a priori hypotheses. However, they also aimed to cultivate an attitude of ‘mindful inquiry’, i.e. to notice, accept and transcend the influence of their own beliefs, knowledge and experiences on interview conduct, interpretation and analysis [24].

**Table 1:** Author Reflexivity.

	Author 1	Author 2	Author 3	Author 4
**Professional role**	Health services researcher	Health services researcher and expert by experience	Health services researcher	Health services researcher and consultant psychiatrist
**Role in the research**	Interviewer	Supporting data analysis	Supporting data analysis	PhD supervisor
	Lead analyst			
**Potential influences on interview conduct or analysis**	Established relationships with interviewees	Personal experience of receiving DBT in the service being evaluated	Familiarity with extant mental health services research literature	Good working relationships with the DBT service
	Good working relationships with the DBT service			Lead on randomised controlled trial evaluation of DBT
	Lead on quantitative evaluation of process-outcome links in DBT			Familiarity with extant DBT research literature
	Familiarity with extant DBT research literature			

### Materials

The topic guide for the interviews was developed in collaboration with DBT clients and therapists, and was modified, following piloting, to reflect areas which emerged as important in early interviews. The topic guide was semi-structured, with key questions followed by suggested probes for further exploration [22]. The order and wording of questioning was flexible in response to the interviewee’s language and the natural course of the conversation [25, 26]. The guide prompted interviewers to explore participants’ experiences of learning, using and benefiting from the skills, what factors had acted as barriers to this process and how they had overcome them. The topic guide is available as supplementary material (S1 Topic Guide).

### Thematic Analysis

The analysts (KB, LC and SS) took a critical realist approach to the data, viewing participants’ accounts of their experiences as grounded in reality, but acknowledging the influence of subjectivity and the social context on data collection and analysis [27]. The interview data was analysed inductively using thematic analysis [28, 29]. Analysis and data collection occurred concurrently in an iterative process whereby early analysis informed conduct of subsequent interviews [23]. The analysts first coded the data i.e. in each interview, sentence(s) of potential relevance to the research questions were tagged in MAXQDA software [30] using brief phrases (‘codes’) that summarised the content [28, 29]. Once all interviews had been coded, credibility checks were conducted by a researcher external to the study team, who independently coded a randomly selected ten percent of the transcripts against the code list. Any discrepancies in coding were discussed and used to modify the list of codes until all raters agreed on code application. The codes were sorted into preliminary themes and sub-themes, which were repeatedly reviewed and refined by all analysts to maximise internal homogeneity and external heterogeneity, until all analysts agreed that the themes and sub-themes accurately reflected the overall ‘story’ told by the data [29]. Once the themes and sub-themes had been finalised, all analysts worked together to develop an analytic narrative for each sub-theme, which summarised what participants had said in each sub-theme and encapsulated the analysts’ interpretation of what each sub-theme meant in relation to the research questions [29].

### Sub-Group Comparisons

The proportion of participants endorsing each sub-theme was compared between treatment completers and dropouts using Fisher’s Exact Test.

### Quality Assurance

In conducting and reporting the results of the study, Elliott and colleagues’ guidelines for qualitative research [31] and the consolidated criteria for reporting qualitative research (CORE-Q) [25], were adhered to.

### Ethics Statement

This qualitative study was approved on 18/02/2009 by the Camden and Islington Research Ethics Committee as a substantial amendment to the DIALECT trial (ref: 07/H0722/98). Participants provided written informed consent to participate. Only participants with capacity to consent were included, after checking they had understood the information provided.

## Results

The analysts derived themes and sub-themes under two key domains pertinent to the research questions: Participant Experiences of Barriers to Skills Training (Domain 1), and Participant Experiences of Overcoming Barriers to Skills Training (Domain 2). Examples of verbatim quotes from participants coded under each sub-theme are given in Tables 2 to 5. Additionally, for each sub-theme an analytic narrative is presented, encapsulating the authors’ interpretation of the meaning of each sub-theme in relation to the research questions.

**Table 2:** Supporting Quotes for Theme 1: Difficulties Learning the Skills (N = 34).

Sub-theme	Examples of Supporting Quotes
Sub-theme 1 Anxiety during the skills groups (N = 25)	“I wouldn’t ask for help cos I was shy and withdrawn and… and they used to say ‘Do you all understand it? I just used to say ‘Yeah’” *[Participant 9*, *DBT completer]*.
	“I didn't take nothing in, everything just went over my head……All I can put it down to is that I didn’t feel comfortable in the group and I didn't wanna be in there, so…… I just couldn’t understand it. I just wanted to get out of the room, that’s all I was thinking of, is getting out of the room” *[Participant 11*, *DBT dropout]*.
	“You’re worried about, you’ve got your homework wrong when you go to the group. And they tell you off in the group, they don’t actually tell you off by yourself” *[Participant 21*, *DBT dropout]*.
	“It was like a child being at school…. And school to me is a terrifying thought….. the more authoritative they were, the more that I was convinced it was school……and I was getting more and more agitated” *[Participant 22*, *DBT dropout]*.
	“I think you have to write off the first couple of classes because it’s very much overwhelming. You’re like, ‘Oh my god I’m a new person, what am I doing here?’ I don’t understand it’. And you’ve just got too much going on to focus on what is being taught anyway” *[participant 25, DBT completer]*.
	“When I finally got there it was still quite shocking. A bit much……Because it’s such a small room as well. And there was loads of people round the table……I felt kind of a bit claustrophobic and nervous…… It’s kinda like a daze, and you don’t remember it……You’ve still got worry thoughts, still in your sad… your mood’s still there and stuff, and you’re still in that frame of ‘Is this gonna work?’ “or ‘I’m never gonna feel like people that have finished’” *[participant 29, DBT completer]*.
Sub-theme 2 Difficulties with the presentation of the skills material (N = 25)	“There is a lot of information…there is a lot coming at you …… the Americanisation of some of the stuff in here is a bit… a bit tricky sometimes to actually um, believe… it sometimes feels like some of the technical words are a bit off-putting… possibly making the language more accessible would make it seem less threatening” *[participant 6, DBT completer]*.
	“Day one I was completely thrown ……you just think, what on earth is ‘mindful’? There’s jargon for you to get to grips with from day one.” *[participant 12, DBT completer]*.
	“They should have explained more of the skills to you instead of big words …..they should shorten it down a bit more…. Not some big posh word where you don’t understand,… it’s very difficult when you don’t understand,” *[participant 21, DBT dropout]*.
	“I don’t understand them……ACCEPTS……Each letter forms something, a word……And in each one, I don’t—I can’t quite grasp the idea of it—of doing it” *[participant 29, DBT completer]*.
	“It’s difficult to translate the way—the DBT language and the way they want you to speak—into normal interaction…… It’s like the jargon…It took me quite a while [….] to understand” *[participant 32, DBT completer]*.
	“The DEARMAN…… I get a little confused with the acronym…and I forget some of the points”*[participant 40, DBT completer]*.

**Table 3:** Supporting Quotes for Theme 2: Difficulties Putting the Skills into Practice (N = 32).

Sub-theme	Examples of Supporting Quotes
**Sub-theme 2.1 Loss of control (N = 26)**	“When I’m in a state, perhaps that’s gonna be the last thing on my mind……I don’t think, ‘Ooh right, I’ll self-soothe’, or, ‘Ooh, use my wise mind’, I just can’t…I feel like my head gets sort of shut in and that’s it” [Participant 1, DBT completer].
	“It's difficult to stop and think, ‘Ooh I should be thinking of… I should be doing this one mindfully’….when you’re feeling really emotional—it’s harder to try and bring yourself together enough to use them…..when I’m sort of in an emotional turmoil inside me” *[Participant 2*, *DBT completer]*.
	“Sometimes……you can’t think about your skills, you’re just overwhelmed….you forget” *[Participant 13*, *DBT dropout]*.
	“When I am really sad or really emotional, nothing comes into my mind then. It’s just… all I can focus on is that feeling. I can’t think this or think that. Nothing sort of… just blackness, that’s all I kind of see” *[Participant 22*, *DBT dropout]*.
	“[The insomnia] really did take a toll on my practising the skills, definitely…..it wouldn’t even come into my head at the end…, I can’t even think of what I’m doing wrong or what is right I can’t think of anything.” *[Participant 36*, *DBT dropout]*.
	“Sometimes I get too emotional and I just can’t use [the skills]. It just gets above that line and I just can’t. No matter what I do I just got to go with it, burst into tears or whatever ‘cause I just can’t stop it”. *[Participant 37, DBT completer]*.
**Sub-theme 2.2 Negative thoughts about the skills (N = 32)**	“Sometimes when I’ve had a relapse I think, ‘It’s too hard, it's too hard to think of everything…there’s too much to think of and it's too much at once, and it's not gonna help.’” *[Participant 2, DBT completer]*.
	“I tried so many times to use the new skills when I felt myself in a situation like… but every time I tried, it failed, so I just gave up trying……I said to myself, ‘This ain’t working, so it ain’t worth trying no more.’*[Participant 11, DBT dropout]*.
	“Learning those skills is gonna be scary, it’s gonna make you very nervous, agitated and it’s gonna be damn hard. So somebody like me is instantly gonna go into your own comfort of what you’ve made yourself to comfort yourself, to stop yourself getting agitated, to stop yourself getting nervous. You’re gonna do it your way again” *[Participant 22, DBT dropout]*.
	“Sometimes I get a little angry … ‘cause I think why do I want all these skills, I don’t want to have to use them around people—nobody else has to use them, why should I?” *[Participant 32, DBT completer]*.
	“I was sick of hearing like ‘What distress tolerance are you going to do?’ I felt like’ I don’t want to do any fucking distress tolerance stuff like, I can’t!…Obviously you’re not understanding how distressed I am and this isn’t going to cover it, like this doesn’t even come close!" *[Participant 33, DBT completer]*.
	“I was just so pissed off with everything that I made sure I didn’t use my skills at all…I just threw all the skills out of the window. Because I just didn’t want to do anything…I’d just had enough.” *[Participant 36, DBT completer]*.

**Table 4:** Supporting quotes for Theme 3: A Personal Journey to a New Way of Life (N = 32).

Sub-theme	Examples of Supporting Quotes
**Sub-theme 3.1 A commitment to keep working towards change (N = 32)**	“It was a journey of going up and down, up and down… it’s trying to train yourself to do things that you don’t do……Sometimes you just get fucked off with trying, that you just can’t be assed anymore. …. Then you can kind of look back and think, ‘No, it did help’. And then you start again.” *[Participant 18*, *DBT completer]*.
	“After three weeks I didn’t understand, I said ‘I’m not going on’…. And then after that I just started getting into it… so I recommend that anyone goes and just give it your best shot. Don’t give up too quickly.” *[Participant 21*, *DBT dropout]*.
	“I remember getting quite upset because I’m thinking to myself, ‘I'm relying on this and I don’t have a clue what they’re going on about.’ …. but you just stick with it, and obviously the more times you go, the more familiar you get with the group.” *[Participant 25*, *DBT completer]*.
	“Mindfulness is a skill that you need to practise…. it got better incrementally… little steps” *[Participant 31*, *DBT dropout]*.
	“[At the beginning I] didn’t understand it I suppose, just didn’t want to do it … I wouldn’t do my homework … but now, I just do it because I want to do it…. Because I know that I want to feel better” *[Participant 36*, *DBT completer]*.
	“I was weaned off [self-harm] though! I didn’t just suddenly quit. I couldn’t do that—I had to try skills, and then maybe I’ll stumble here and there and I’ll go back to it, and then I’ll have to try again” *[Participant 39*, *DBT completer]*.
**Sub-theme 3.2 Making the skills my own (N = 13)**	“I began to have favourite skills and less favourite ones and as soon as you start choosing what you like and what you don't like, it’s not something scary anymore. It’s almost your friend, your thing to turn to” *[Participant 12*, *DBT completer]*.
**Sub-theme 3.3 Using the skills becomes automatic (N = 18)**	“Because I was so focused at trying to be perfect at it and making it making it work just as it should by the book, it was hindering the effectiveness of it … Since I've left and I've just had the skills in my head and in my mind, they just sort of click into place whenever I want to use them….. I’m just, I’m not so focused on doing everything exactly as it says in the manual”. *[Participant 19*, *DBT dropout]*.
	“When you’re in the group sessions… in your mind you kind of learn to associate what the lesson is about with what your own life, so then you can make notes to say ‘Oh like this’. And you read back through, so yeah, you understand exactly what it means to you” *[Participant 26*, *DBT completer]*.
	“Some of the skills are becoming second nature… I’m doing it sometimes without thinking about it.” *[Participant 2*, *DBT completer]*.
	“It becomes automatic and you use them so frequently that it just becomes a part of your day to day life” *[Participant 12*, *DBT completer]*.
	“The good thing about DBT is that skills become ingrained. Over that year, the more you do it the more it becomes a part of you, till you’re doing it without knowing you’re doing it……” *[Participant 35*, *DBT completer]*.

**Table 5:** Supporting quotes for Theme 4: An Environment that Supports Change (N = 31).

Sub-theme	Examples of Supporting Quotes
**Sub-theme 4.1 The skills group (N = 25)**	“I find the groups where I learn… where sort of everything sinks in a bit more, are the ones that have been a bit lighter and there's been a bit of laughter.”*[Participant 2*, *DBT completer]*.
	“There are certain teachers that will work round and ask people for examples, and I think that needs to be kind of maintained to keep people’s attention… just getting the interaction as opposed to someone standing there and just reading stuff out.” *[Participant 25*, *DBT completer]*.
	“Sometimes they put their personal experience in as well which I think is helpful…… It stops you feeling quite so much like a schoolchild; makes it more of an interactive experience…… It stops you as well from feeling disconnected from the rest of humanity rather than just being sort of… someone who always has problems” *[Participant 31*, *DBT dropout]*.
	“I was like 'This is never gonna work'… [But] other people were saying it worked……It was like ‘Nothing else has worked, if people are saying this shit works, I might as well try it.’” *[Participant 39*, *DBT completer]*.
	“Other people would come and be like ‘Oh, this happened, this situation was happening and this is how they acted’……You can always think about situations of your own that are similar to that, and then you think ‘ Ah I guess I could do it that way’, y’know……I can take more from someone’s personal experience than if they just give me sometimes a hypothetical question.” *[Participant 40*, *DBT completer]*.
**Sub-theme 4.2 The individual therapist (N = 23)**	“When I didn’t [understand], I’d go to see my one to one therapist, and he would explain them to me, and they would become clear…I understood after I went to him” *[Participant 13*, *DBT dropout]*.
	“Where I felt really sad and felt to self-harm, we would talk about what could have happened differently before that thought came that you know like, Distress Tolerance, what you could have done to…to change that situation, different distress techniques, tolerance techniques that you could use” *[Participant 19*, *DBT completer]*.
	“In the role play, right, you’ve got nothing to lose, you know that this is pretend. But you can come up with every single scenario, and every single ‘He might say this, or he might say this, or this might happen or this might happen—so what will you do if this happens?’ And so you know what to expect.” *[Participant 39*, *DBT completer]*.
**Sub-theme 4.3 Friends and family (N = 13)**	“Cause [Partner]’s also gone on the family DBT group. Um, she’ll sort of say… ‘This is a fact’. Or ‘What you… what you’re saying is not based on fact, give me a fact’. And then it’ll just… it just seems to take the heat out of the argument somewhat” *[Participant 4*, *DBT completer]*.
	“I spoke to [Partner], my other half, about what I learnt… in turn sometimes when I was maybe lacking in using my skills, he would be able to give me that kick up the backside” *[Participant 25*, *DBT completer]*.

### Domain 1: Participant Experiences of Barriers to Skills Training

#### Theme 1. Difficulties learning the skills: too much to take in

Participants reported difficulties learning the skills linked to two key sub-themes: Anxiety during the skills groups (Sub-theme 1.1.) and Difficulty with the presentation of the skills material (Sub-theme 1.2). Examples of supporting quotes for each of these sub-themes are given in Table 2 below.

*Sub-theme 1*.*1 Anxiety during the skills groups*: For twenty-five participants, attending the skills groups was at times an anxiety-provoking experience. Elements leading to anxiety included worrying about being judged by the therapists or other group members, judging themselves when they did not understand the material, or concern that the treatment would not work. For a few, the therapists were perceived as authoritarian and strict, whilst the school-like environment triggered traumatic childhood memories. This interfered with participants’ ability to learn the skills in various ways, including interfering with their ability to concentrate on the material being taught, feeling too embarrassed to ask for help when they did not understand, or having urges to escape the room or end therapy.

*Sub-theme 1*.*2 Difficulties with the presentation of the skills material*: Twenty-five participants reported that certain aspects of the way the skills material was presented made it difficult to understand and learn. These included being faced with a lot of information presented at a rapid pace, the use of specialist DBT jargon that was difficult to understand, and the use of acronyms which were difficult to remember.

#### Theme 2. Difficulties putting the skills into practice: overwhelming emotions

Thirty-two of the forty participants explained that feeling overwhelmed by intense emotions at times prevented them from putting the skills into practice, by leading them to perceive a loss of control over their behaviour (Sub-theme 2.1) or leading to negative thoughts about using the skills (Sub-theme 2.2). Examples of supporting quotes for each of these sub-themes are given in Table 3.

*Sub-theme 2*.*1 Loss of control*: Twenty-six participants explained that at times intense emotions seemed to ‘take over’ their mind, such that they felt a loss of control over their own thoughts and behaviour. They explained that their emotions could overwhelm their thoughts to such an extent that it felt like *“blackness”* [Participant 22, DBT dropout] or a *“massive fog”* [Participant 25, DBT completer], and as a result it was very difficult to remember to use the skills or to think about using them.

*Sub-theme 2*.*2 Negative thoughts about the skills*: Thirty-two participants explained that at times they would have negative thoughts about the skills, such as thinking that they did not want to try to use them, and that using them was too difficult, pointless or would not work. They emphasised that continually trying to use the skills in the face of distressing experiences could be a difficult struggle and was often emotionally exhausting. For some, negative thoughts were linked to feelings of defiance or rebellion against having to use the skills. For others, the thought of using the skills was highly anxiety-provoking, forcing them to confront things they would rather avoid, take risks and let go of their ‘safe’ coping behaviours.

### Domain 2: Participant Experiences of Overcoming Barriers to Skills Training

#### Theme 3. A personal journey to a new way of life

Participants described the challenging journey they had undergone to overcome initial difficulties in learning and using the skills. As they committed and re-committed to keep working towards change in their lives, (sub-theme 3.1), they gradually began to personalise the way they used the skills (sub-theme 3.2), and to find that the skills became an automatic part of their behavioural repertoire (sub-theme 3.3). Supporting quotes are shown in Table 4.

*Sub-theme 3*.*1 A commitment to keep working towards change*: Almost all participants (N = 32) emphasised that overcoming barriers to learning and using the skills was a gradual journey, requiring a continual process of committing and re-committing to working towards change. Participants did this by continuing to attend the group, even when at first they did not understand what was being taught, and did not believe it would help them. They practised using the skills again and again in their daily lives, even when at first it was difficult or did not seem to work. When they felt tempted to give up, they re-committed to maintaining their efforts by reminding themselves of how much they wanted to change their lives, and also of the progress they had made.

*Sub-theme 3*.*2 Making the skills my own*: Over the course of DBT, a minority of participants (N = 13) gradually derived their own personal meanings and interpretations of the skills, and learnt to concentrate on using the skills that worked best for them and their personal circumstances. For some, this personalisation was important in enabling them to let go of rigid, perfectionistic and self-critical thinking about their skills use.

*Sub-theme 3*.*3 Using the skills becomes automatic*: For nearly half of the participants (N = 18), the end result of this journey was that eventually they became able to use the skills automatically without having to think about it. For a few, the DBT skills became so ingrained that they referred to them as “part of” themselves, an integral aspect of their identity.

#### Theme 4. An environment that supports change

The second theme concerns the beneficial effect of support from others in learning and using the skills: the skills group (sub-theme 4.1), the individual therapist (sub-theme 4.2) and friends and family (sub-theme 4.3). Supporting quotes are shown in Table 5.

*Sub-theme 4*.*1 The skills group*: Many participants (N = 25) found the skills group to be an environment that supported them in overcoming difficulties with learning and using the skills. Participants emphasised the importance of being able to learn from each other’s understanding of the skills and experiences using them, and of being encouraged by more experienced group members to persevere through doubts and difficulties. For some, learning from the group therapists about how they used the skills to deal with problems in their own lives gave them a sense of connectedness and made learning the skills a less isolating experience; less of a ‘them and us’. Participants found learning much easier when there was a fun, light-hearted atmosphere in the group, with everybody included. This was created through the interactions between group members and through the efforts of the therapists to teach in a fun way using multiple formats, and to keep checking that everybody understood.

*Sub-theme 4*.*2 The individual therapist*: Many participants (N = 23) reflected on the importance of their individual therapist in supporting their skill use. The individual therapist was able to explain things they had not understood during group skills training, suggest skills to try during telephone skills coaching and role play skilful behaviour. If target behaviours, e.g. self-harm, had occurred in the previous week, they could use chain analysis to help the participant see where they could have used the skills.

*Sub-theme 4*.*3 Friends and family*: A minority of participants (N = 13) reported that their friends and family had supported their skill use in a number of ways: by discussing the skills with them, helping them understand difficult concepts, encouraging them to use the skills, or even using the skills themselves.

### Subgroup Comparisons

A comparison of the proportions of participants endorsing each sub-theme in the treatment completers versus treatment dropouts sub-groups is shown in Table 6 below. Dropouts were more likely than completers to have experienced anxiety during the skills groups as a barrier to learning the skills (p = 0.05) and were less likely to report committing to persisting in working towards change (p = 0.02), personalising their use of the skills (p = 0.07) or becoming able to use the skills automatically (p = 0.02). They were also less likely to report experiencing the skills group (p = 0.03), their individual therapist (p = 0.01) or friends and family (p = 0.07) as supporting their skills training.

**Table 6:** A Comparison of Theme Endorsement by Treatment Completers and Treatment Dropouts.

Domain	Theme	Sub-theme	Completers (N = 27)	Dropouts (N = 13)	Fisher’s Exact p value
Experiences of Barriers to Skills Training	Difficulties Learning the Skills: Too Much to Take In	Anxiety during the Skills Groups	14 (52%)	1 (85%)	0.05
		Difficulties With the Presentation of the Skills Material	19 (70%)	6 (46%)	0.13
	Difficulties Putting the Skills into Practice: Overwhelming Emotions	Loss of Control	17 (63%)	8 (62%)	0.60
		Negative Thoughts about the Skills	22 (81%)	11 (85%)	0.60
Experiences of Overcoming Barriers to Skills Training	A Personal Journey to a New Way of Life	A Commitment to Keep Working Towards Change	24 (89%)	7 (54%)	0.02
		Making the Skills My Own	12 (44%)	2 (15%)	0.07
		Using the Skills Becomes Automatic	15 (56%)	2 (15%)	0.02
	An Environment that Supports Change	The Skills Group	20 (74%)	5 (38%)	0.03
		The Individual Therapist	24 (89%)	5 (38%)	0.01
		Friends and Family	12 (44%)	2 (15%)	0.07

## Discussion

### Summary of the Findings

Based on in-depth interviews with 40 clients with BPD who had taken part in a DBT programme, the study aimed to establish what factors clients experienced as barriers to DBT skills training, how clients experienced overcoming barriers to skills training, and how these experiences differed between treatment completers and treatment dropouts. A thematic analysis of participants’ reported experiences found that key barriers to learning the skills were anxiety during the skills groups and difficulty understanding the material. Key barriers to using the skills were overwhelming emotions which left participants feeling unable or unwilling to use them. Key ways in which participants reported overcoming barriers to skills training were by sustaining their commitment to attending therapy and practising using the skills, personalising the way they used skills, and practising them so often that they became an integral part of their behavioural repertoire. Participants also highlighted the importance of other skills group members, the group therapists, individual therapists, friends and family for explaining the skills and exemplifying how to use them, whilst making skills training an enjoyable and collaborative process. Treatment dropouts were more likely than completers to describe anxiety during the skills groups as a barrier to learning, and were less likely to report overcoming barriers to skills training via the key processes outlined above.

### Clinical and Research Implications of the Findings

Due to the subjective nature of qualitative findings, which are based on participants’ interpretations of their experiences which are then in turn interpreted by the analysts, the present study can only raise tentative suggestions for clinical implications and hypotheses for testing in further research. These are outlined below.

#### Monitoring problematic thoughts and feelings

Experiences of anxiety during the skills group, difficulties with the skills material, a perceived loss of control over behaviour, and negative thoughts about using the skills, should all be treated as Target 2 “therapy-interfering behaviours” [5], if they interfere with clients’ ability to learn and use the skills. However, these experiences may not always be apparent to therapists. It could perhaps be helpful to add “Anxiety during the skills groups”, “Difficulties with the skills material”, “Feeling out of control” and “Negative thoughts about the skills” to the Diary Card to enable the individual therapist to monitor, explore, validate and target them as necessary.

#### Skills coaching

Individual therapists may find it helpful to suggest various DBT skills to clients experiencing problematic levels of anxiety during the groups, perceived loss of control or negative thoughts about the skills. These include: considering pros and cons of using the skills to cope versus acting on their urges; ‘acting opposite’ (e.g. actively participating in group despite urges to withdraw, using the skills despite negative thoughts about them); recognising and letting go of judgemental thoughts about group or the skills; challenging negative thoughts about the group or using the skills; ‘cheerleading’ (thinking encouraging thoughts); ‘Cope Ahead’ (e.g. imagining oneself coping with feeling anxious during group); and radical acceptance (e.g. accepting that feelings of anxiety are part of life and must at times be endured) [4, 32]. Clients who report becoming so overwhelmed by their emotions that they lose control and “cannot” use the skills, may lack awareness of early warning signs that their emotions are escalating. Therapists may find that coaching clients to scan their bodies regularly could allow the client to notice early indicators of emotional arousal and intervene by using the skills, before reaching the point of feeling overwhelmed [4, 32].

#### Tailored interactional styles

Group therapists may find it helpful to modify their interactions with clients for whom anxiety during the skills groups has been identified as a barrier, by taking a gentler approach focussed on validating clients’ answers to questions and feedback on homework practice [32].

#### Presentation of the skills material

Group therapists may find it helpful to repeatedly define any specialist terminology used (e.g. ‘mastery’, ‘radical acceptance’). Participants in the present study found it particularly helpful when group therapists made the skills teaching interactive and fun, using a variety of teaching techniques including group exercises, diagrams, role plays and self-disclosure.

#### Reinforcing motivation and commitment

Therapists may find it helpful to draw on Linehan’s suggested strategies for reinforcing clients’ commitment to maintaining their efforts to learn and use the skills [5]. These include evaluating the pros and cons of making such a commitment, playing devil’s advocate, using the ‘foot in the door’ technique, highlighting prior commitments made by the client, and cheerleading the client.

#### Suggestions for further research

Further research could monitor state anxiety before, during and after the skills groups and test whether increases in state anxiety during the group predict dropout. If found to be linked to dropout, a feedback system could be implemented whereby state anxiety measures are routinely administered during DBT skills groups, and clients reporting increases in anxiety during the groups are ‘flagged up’ to their individual therapists as being potentially at risk of dropout. This feedback approach has been shown to improve outcome in mixed diagnosis groups but has not yet been tested in personality disorder [33, 34, 35, 36].

Participants in the present study highlighted the role of support from other group members in overcoming barriers to skills training. Further research could test whether assigning new group members a skills coaching ‘buddy’ from amongst the more experienced group members, to provide encouragement and share their own experiences of learning and using the skills, could help reduce dropout.

### Limitations

The interviews focussed only on the skills training element of DBT, preventing exploration of other aspects of the treatment that may have contributed to treatment dropout. Additionally, participants who dropped out of treatment may have retrospectively adjusted their recall of their experiences in line with their outcome, thus being more likely to recall learning and using the skills in a negative light. The samples were small and not randomly selected—hence, differences between dropouts and completers are not robust findings but can yield plausible hypotheses for testing in further research.

### Conclusion

The present study was able to produce novel insights on barriers to DBT skills training and how they can be overcome, through analysis of the rich, experiential data provided by qualitative interviews with DBT participants. Although the findings are subjective and require replication, they could be valuable for generating ideas on how therapists can help their clients overcome barriers to skills training, and also hypotheses for testing in further research.

## Supporting Information

S1 Topic Guide Topic Guide for Qualitative Interviews. (DOC)
