# Supplementary material for: Overcoming Barriers to Skills Training in Borderline Personality Disorder: A Qualitative Interview Study
Source: PLoS One. 2015 Oct 14;10(10):e0140635. doi: 10.1371/journal.pone.0140635 (PMC4605586; doi:10.1371/journal.pone.0140635)
Supplement: S1 Topic Guide — (DOC) [file pone.0140635.s001.doc]

**S1 – Interview Topic Guide**

**Interviewer Instructions:**

**Questions in CAPITALS are key questions to be asked of all participants.**

**All other questions/ statements are suggested prompts only – use them only if the interviewee does not bring them up spontaneously, and phrase them in your own words and appropriately to the context of what the interviewee has been saying.**

Interview Suggested Content

1) HOW LONG HAVE YOU BEEN DOING DBT? Group and individual?

2) HOW HAS YOUR MENTAL HEALTH CHANGED SINCE YOU STARTED DBT? In what way? What hasn’t changed?

(probe self-harm, suicidality, and emotional instability especially)

3) WHAT SKILLS HAVE YOU BEEN TAUGHT? WHAT DO YOU THINK OF THEM? HAVE THE SKILLS HELPED YOU? HOW?

*Can you explain some more? What do you mean? Give me an example?*

4) HOW EASY HAVE YOU FOUND IT TO LEARN THE SKILLS YOU’VE BEEN TAUGHT?

Has anything helped you to learn the skills?

Has anything got in the way of learning the skills? Have you overcome these difficulties, and if so, how?

*Can you explain some more? What do you mean? Give me an example?*

5) HOW DO YOU USE THE SKILLS?

Which skills do you use the most?

*Why? Can you explain some more? What do you mean? Give me an example?*

When do you use the skills?

*Can you explain some more? What do you mean? Give me an example?*

How easy have you found it to use the skills?

*How has that changed since starting therapy? Can you explain some more? What do you mean? Give me an example?*

Is there anything that has helped you to use the skills?

*Can you explain some more? What do you mean? Give me an example?*

Is there anything that has got in the way of using the skills?

*Can you explain some more? What do you mean? Give me an example?*

Have you overcome these difficulties, and if so, how? *How has that changed since starting therapy?* *Can you explain some more? What do you mean? Give me an example*

Is there anything that has stopped the skills from being more helpful to you? *Can you explain some more? What do you mean? Give me an example?*

Have you overcome these difficulties, and if so, how? *Can you explain some more? What do you mean? Give me an example*

Is there anything that has made the skills more helpful for you? *Can you explain some more? What do you mean? Give me an example?* Has that changed since starting therapy?

6) IS THERE ANYTHING ELSE YOU’D LIKE TO SAY ABOUT YOUR EXPERIENCE OF LEARNING AND USING THE SKILLS?

*Can you explain some more? What do you mean? Give me an example?*

7) IS THERE ANYTHING ELSE THAT HAS HELPED YOU ACHIEVE CHANGES IN YOUR MENTAL HEALTH?

*Can you explain some more? What do you mean? Give me an example?*

8) IS THERE ANYTHING ELSE THAT HAS BEEN A BARRIER TO MAKING CHANGES IN YOUR MENTAL HEATLH?

*Can you explain some more? What do you mean? Give me an example?*

9) ANYTHING ELSE YOU WANT TO ADD?
